# Dataset on the assessment of water quality and water quality index of Ubogo and Egini rivers, Udu LGA, Delta State Nigeria

**DOI:** 10.1016/j.dib.2018.06.053

**Published:** 2018-06-27

**Authors:** A.S. Ejoh, B.A. Unuakpa, F.H. Ibadin, S.O. Edeki

**Affiliations:** a Department of Biological Sciences, Covenant University, Ota, Nigeria; b Department of Animal and Environmental Biology, University of Benin, Nigeria; c Department of Biological Sciences, Mountain Top University, Makogi Oba, Nigeria; d Department of Mathematics, Covenant University, Ota, Nigeria

**Keywords:** Physico-Chemistry, Water quality, Water quality index, Decomposiion method

## Abstract

The article contains datasets built on a baseline study on the selected physicochemical parameters and Water Quality Index of Ubogo and Egini Rivers, Delta State, for a period of six months, spanning the time frame between February - July, 2010. Within this space, six stations were shared equally among the two rivers using the three communities they flow through as guide, and water samples were collected on monthly basis from these stations. The objectives include determination of the spatial variations and background concentrations of the selected physicochemical parameters. Sixteen physicochemical parameters were analyzed in the water. Current velocity, air and water temperature were determined in-situ; the rest physicochemical parameters were determined via adopting standard methods. Apart from turbidity, the values of the rest physical parameters – air and water temperature, TDS showed significant difference (p < 0.05) across the stations.

**Specifications Table**

**Table:** 

Subject area	*Environmental Sciences.*
More specific subject area	*Environmental and aquatic study.*
Type of data	*Tables, Charts and Figures.*
How data was acquired	*Periodic sampling via 0-100*^*0*^*C mercury in glass thermometer and surface floating method.*
Data format	*Raw, Processed and Analysed.*
Experimental factors	Investigation of the water quality, physic chemistry of water and water quality index.
Experimental features	Water quality, physic-chemistry of Water and Water Quality Index of Ubogo and Egini rivers.
Data source location	Egini-Ubogo stream, Delta, Lat 5.45′ – 6.20N, and long 5.24′ – 6°.20׳E Nigeria.
Data accessibility	*Data is within this article.*

**Value of the data**

• The dataset provided in this article reflects the physico-chemical status of the Ubogo and Egini rivers.
• The dataset article discusses the importance of the physico-chemical status and water quality of the Ubogo and Egini Rivers.
• The nature of the dataset can be used for estimating the water quality index of the Ubogo and Egini rivers.
• The dataset will help to determine the effects of chemical contamination in the concerned rivers.
• The information contained herein can be extended to study other rivers in terms of water quality.

## 1 Data

**Fig. 1:**
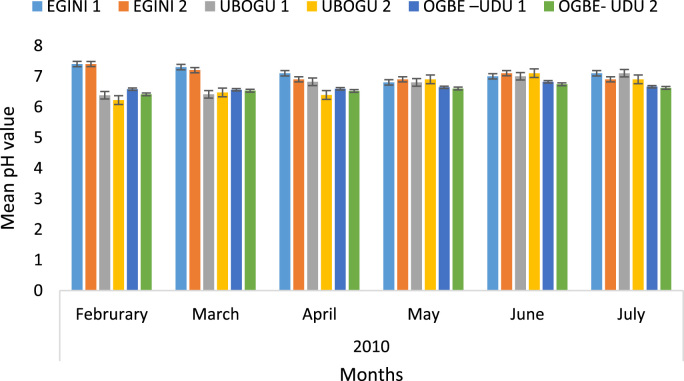
Mean Temporal and spatial variations in PH value.

**Fig. 2:**
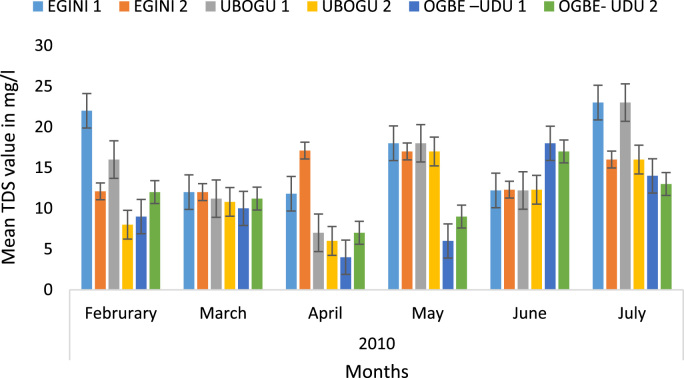
Mean Temporal and spatial variations in Total Dissolved Solid (TDS).

**Fig. 3:**
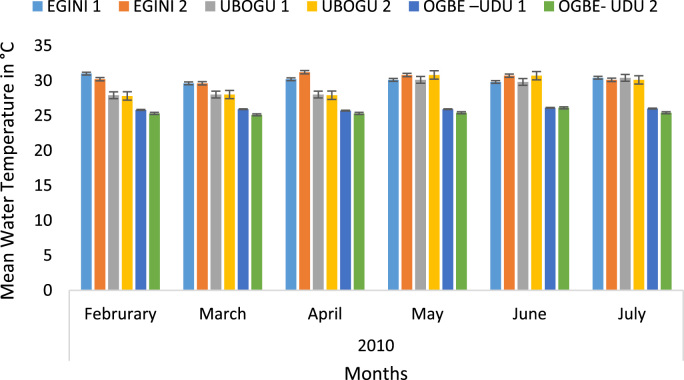
Mean Temporal and spatial variations in water temperature.

**Fig. 4:**
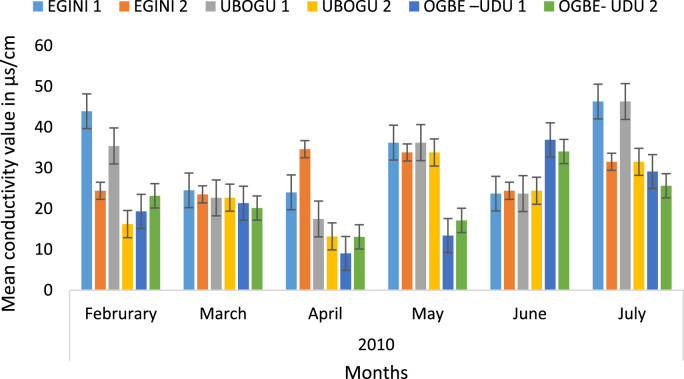
Mean Temporal and spatial variations in conductivity.

**Fig. 5:**
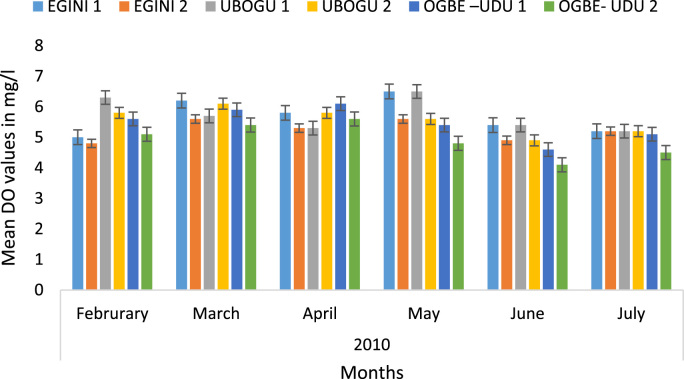
Mean Temporal and spatial variations in dissolved oxygen (DO) values.

**Fig. 6:**
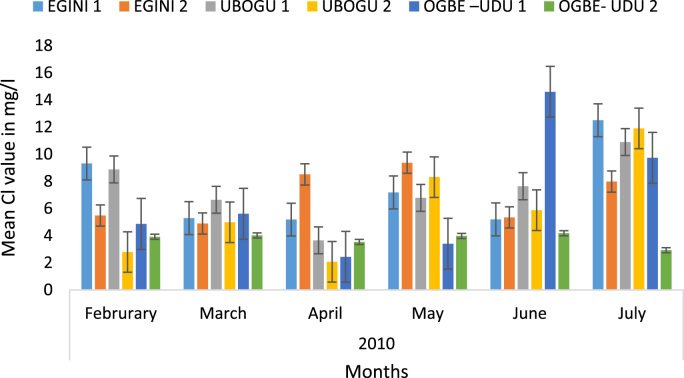
Mean Temporal and spatial variations in CI.

**Fig. 7:**
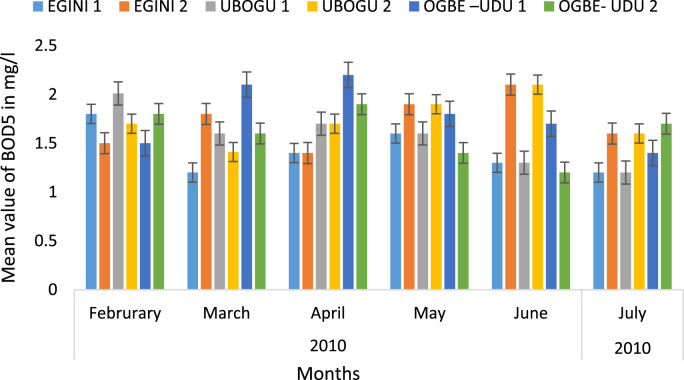
Mean Temporal and spatial variations in BODs.

**Fig. 8:**
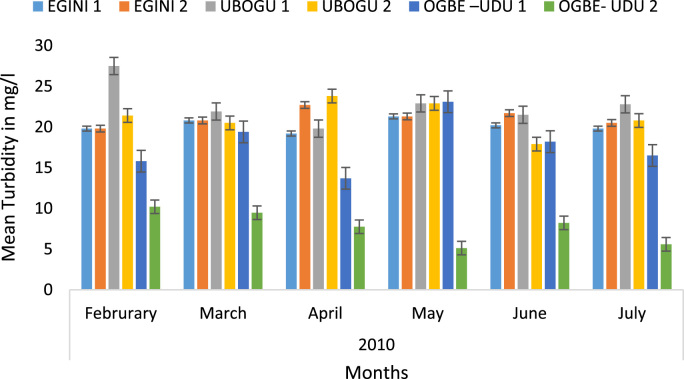
Mean Temporal and spatial variations in Turbidity.

**Fig. 9:**
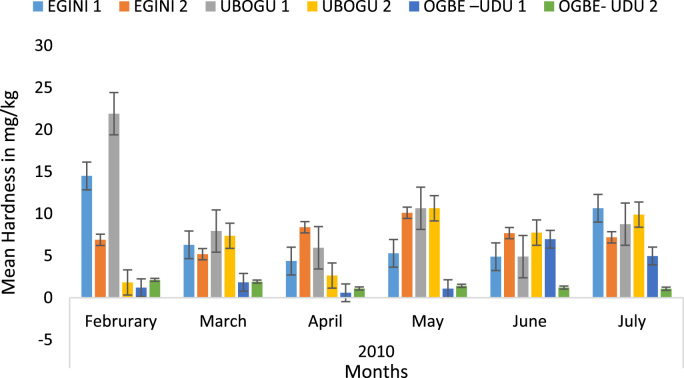
Mean Temporal and spatial variations in hardness.

**Fig. 10:**
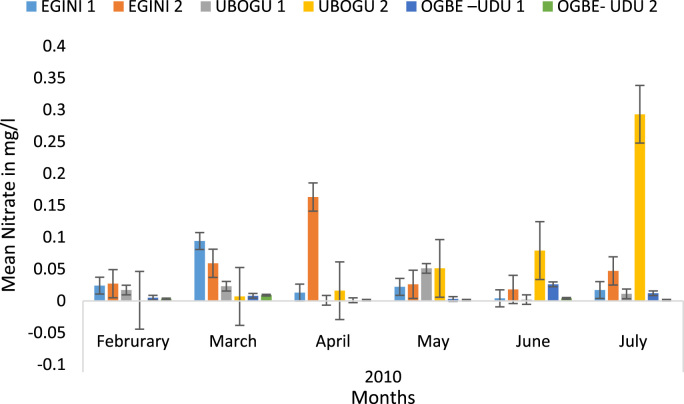
Mean Temporal and spatial variations in nitrate.

**Fig. 11:**
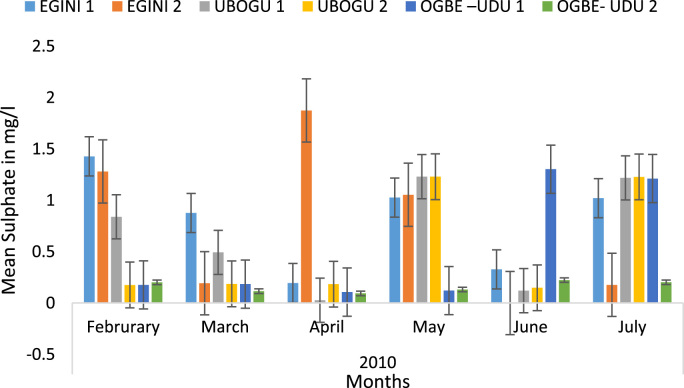
Mean Temporal and spatial variations in sulphate.

**Fig. 12:**
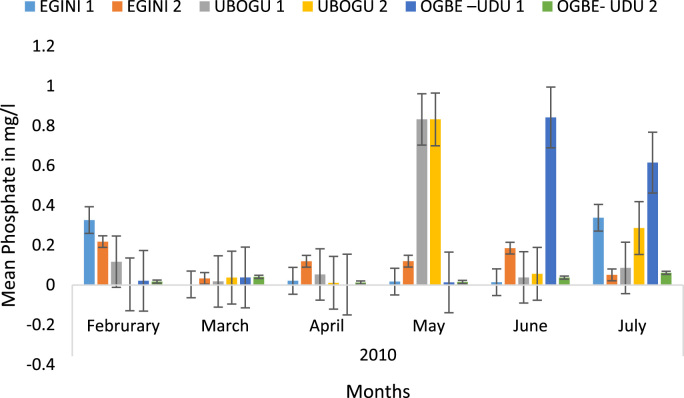
Mean Temporal and spatial variations in phosphate.

**Fig. 13:**
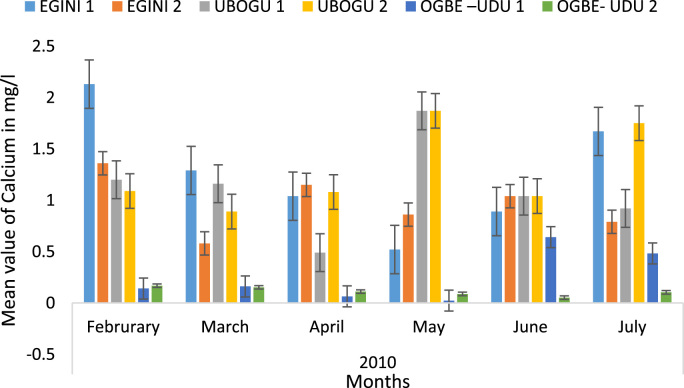
Mean Temporal and spatial variations in calcium.

**Fig. 14:**
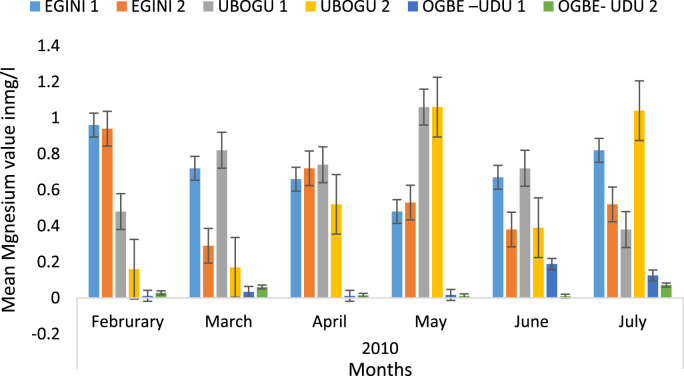
Mean Temporal and spatial variations in magnesium value.

**Fig. 15:**
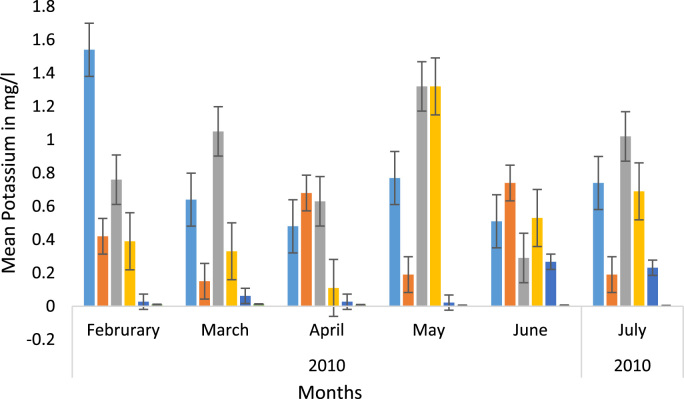
Mean Temporal and spatial variations in potassium.

**Fig. 16:**
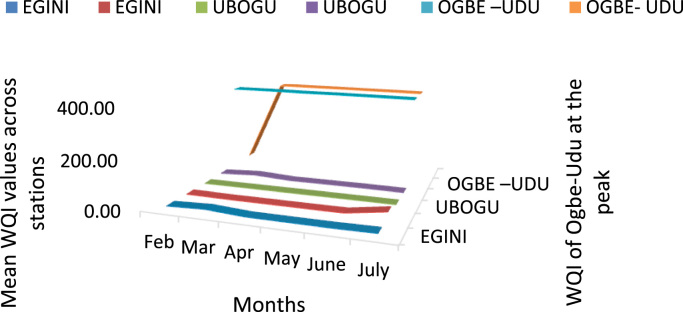
Spatial and monthly variations of WQI of Egini, Ugbogu, Rivers Delta state.

**Fig. 17:**
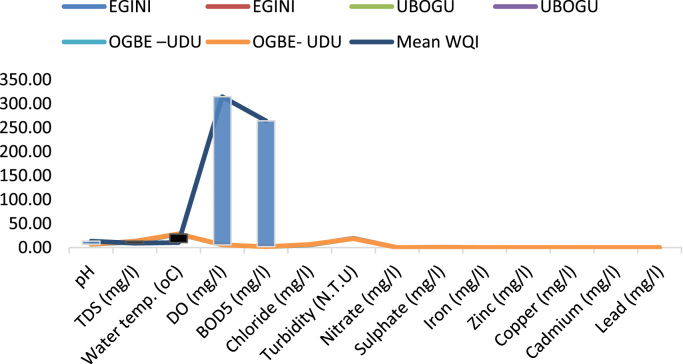
Relationship between WQI and the Physical and chemical parameters at Egini, Ugbogu and Ogbe-Udu, Rivers Delta state.

Here, the climate, vegetation and land use are considered. The study area has distinct climate region located at the position of the inter-tropical convergence zone (ITCZ). The rainy season is from March to October, while the dry season spans from November to February of the following year. Temperature values follow the pattern of variation of relative humidity. The study area consists primarily of tropical rainforest. This is characterized by sparse vegetation in the western region which becomes denser eastwards. The stream is fairly narrow, dark turbid and is flanked by oil palm trees, mahogany trees, raffia palm, and rubber trees along the banks. Farming and fishing are the primary activities that take place in the rivers, while laundry is a secondary human activity on the bank of this stream. In Fig. 1 through Fig. 15, graphical relationship of some variational parameters are displayed. These include: PH value (Fig. 1), Total Dissolved Solid (TDS) (Fig. 2), Water Temperature (Fig. 3), variations in Conductivity (Fig. 4), Dissolved Oxygen (DO) Values (Fig. 5), Cloride(CL) (Fig. 6), Biochemical Oxygen Demands (BODs) (Fig. 7), Turbidity (Fig. 8), Hardness (Fig. 9), Nitrate (Fig. 10), Sulphate (Fig. 11), Phosphate (Fig. 12), Calcium (Fig. 13), Magnesium value (Fig. 14), Potassium (Fig. 15). The relationship between monthly and spatial variations are presented via Fig. 16 in terms of WQI. Fig. 17 shows the link between WQI and the physical chemical parameters at the river-sides. This study was carried out on Egini-Ubogo stream, Delta, located within Latitude 5.45׳ – 6.20N, and longitude 5.24׳ – 60.20׳E. The streams sourced from Ohworode and flows westerly into Okpare creek at Oto-Udu. Okpare creek empties into Forcados River at Okwagbe-Otor.

## 2 Experimental design, materials and methods

In relation to the flow direction, two sampling stations were positioned along Egini River (Egini 1-2), and four along Ubogo River (Ubogu 1-4), two each around Ubogo and Ogbe-Udu community.

### 2.1 Sampling locations

The sampling stations were visited during the sampling period between February and July, 2010. Sampling stations were chosen on the basis of their proximity to facilities, structure or human activities that could potentially affect water quality and biodiversity.

Egini 1: The water current is fairly high. The substratum is clay and mud. The water is dark and turbid. Marginal vegetation is basically rubber trees as well as floating grasses. Human activities noticed during the course of study were primarily fishing, bathing and laundry as well as washing of cars and motor cycles along the bank. This station is at a bridge site. Egini 2: It is located approximately 1km from Egini 1. The water is dark and turbid. The prevalent vegetation is rubber trees and palm trees as well as floating grasses. The substratum is clay and mud. Human activities observed was fishing and bathing.

Ubogo 1: It is at a bridge site. The current velocity is very high. The substratum is a mixture of sand, silt and clay. Marginal vegetation consists of grasses, rubber trees, oil palm and mahogany. Human activities including bathing and laundry as well as washing of motor cycles are very pronounced. Ubogo 2: The water is dark and tubid with a moderate current of flow. The substratum is a mixture of sand, silt and mud. The marginal vegetation consists of rubber trees, oil palm and sparse grasses. The only human activity noticed was fishing. The location is located downstream of Ubogo 1. Ubogo 3: Ogbe Udu is located approximately 3km downstream of Ubogo. The water is dark, turbid and very slow flowing. The substratum is silt, sand and little of muddy from deposited debris. The only human activity noticed is fishing. Ubogo 4: The water is dark and turbid and very slow flowing. The substratum is silt, sand and mud. Fishing was the only human activity observed in Ubogu 4.

### 2.2 Sampling

Bimonthly samples were collected between February and July, 2010. At every point, samples were taken between the hours of 7:00 am and 9:00 am. For each station the water and air temperature were determined in situ using 0-100^o^C mercury in glass thermometer. Water current velocity was determined at each station using surface float method. This involves the floating of a cork material in the lotic water at a given time and the current velocity was calculated in m/s using the formula:

(1) $$c_{v}=\frac{m}{s}$$

where $c_{v}$ implies current velocity, m is the distance, and s is time parameter. Related researches on water quality and its assessment are not limited to the following [1], [2], [3], [4], [5], [6], [7], [8], [9], [10], [11], [12], [13], [14], [15], [16], [17], [18], [19].

### 2.3 Descriptive statistics and analysis of variance (ANOVA)

Basic statistical measurement of central tendency and dispersion was used to characterize the stations in terms of physicochemical conditions. Inter station comparisons were carried out to test for significant differences in the physiochemical conditions using parametric analysis of variance (ANOVA). Duncan multiple range (DMR) tests were performed to determine the location of differences using the computer SPSS 16.0 window application based on Intel ® core ™ i5-3230M CPU@2.60GHz 2.60GHZ. Descriptive statistics and summary of the physico-chemical characteristics of the two rivers are presented in Table 2.1.

**Table 2.1:** Summary of the Physico-Chemical Characteristics of the Egini, and Ubogo Rivers.

		EGINI		UBOGO		OGBE –UDU			
		Egini -1	Egini 2	Ubogo - 1	Ubogo - 2	Ubogo – 3	Ubogo 4		
Parameters	Units	$\overset{¯}{X}$ ± SD (Min-Max)	$\overset{¯}{X}$ ± SD (Min-Max)	$\overset{¯}{X}$ ± SD (Min-Max)	$\overset{¯}{X}$ ± SD (Min-Max)	$\overset{¯}{X}$ ± SD (Min-Max)	$\overset{¯}{X}$ ± SD (Min-Max)	*p*-Value	FMEnv
Air temperature	°C	32.60 ± 0.57 ^bc^ (32.00–33.50)	33.72 ± 0.42 ^a^ (33.00–34.20)	33.18 ± 0.75 ^ab^ (31.80–33.80)	32.15 ± 0.98 ^c^ (31.00–33.50)	29.62 ± 0.49 ^d^ (29.00–30.10)	29.52 ± 0.53 ^d^ (29.00–30.00)	*p* < 0.01	N/A
Water temperature	°C	30.17 ± 0.51 ^ab^ (29.60–31.00)	30.43 ± 0.58 ^a^ (29.60–31.20)	29.03 ± 1.18 ^bc^ (27.90–30.40)	29.22 ± 1.46 ^c^ (27.80–30.80)	25.90 ± 0.14 ^d^ (25.70–26.10)	25.43 ± 0.34 ^d^ (25.10–26.10)	*p* < 0.01	35.00
TDS	mg/l	13.18 ± 5.15 ^ab^ (8.00–22.00)	11.20 ± 0.76 ^b^ (10.00–12.00)	8.82 ± 4.80 ^b^ (4.00–17.10)	14.17 ± 5.27 ^ab^ (6.00–18.00)	14.00 ± 2.73 ^ab^ (12.20–18.00)	17.50 ± 4.42 ^a^ (13.00–23.00)	*p* < 0.05	500.00
Turbidity	N.T.U	19.08 ± 5.78 (10.20–27.50)	18.81 ± 4.65 (9.47–21.90)	17.82 ± 6.07 (7.74–23.80)	19.44 ± 7.06 (5.13–23.10)	17.95 ± 5.03 (8.22–21.70)	17.67 ± 6.26 (5.59–22.80)	p > 0.05	5.00
pH		6.73 ± 0.53 (6.22–7.40)	6.75 ± 0.40 (6.41–7.30)	6.72 ± 0.27 (6.39–7.10)	6.77 ± 0.13 (6.60–6.90)	6.96 ± 0.15 (6.74–7.10)	6.88 ± 0.21 (6.62–7.10)	*p* > 0.05	6.50-8.50
Conductivity	µS/cm	27.07 ± 10.51 ^ab^ (16.23–43.90)	22.48 ± 1.54 ^b^ (20.16–24.50)	18.56 ± 9.36 ^b^ (9.02–34.60)	28.42 ± 10.32 ^ab^ (13.41–36.20)	27.85 ± 5.97 ^ab^ (23.70–36.89)	35.05 ± 8.97 ^a^ (25.62–46.30)	*p* < 0.05	N/A
DO	mg/l	5.43 ± 0.57 ^ab^ (4.80–6.30)	5.82 ± 0.31 ^a^ (5.40–6.20)	5.65 ± 0.31 ^a^ (5.30–6.10)	5.73 ± 0.66 ^a^ (4.80–6.50)	4.88 ± 0.50 ^b^ (4.10–5.40)	5.07 ± 0.28 ^b^ (4.50–5.20)	*p* < 0.01	5.0
BOD_5_	mg/l	1.72 ± 0.20 (1.50–2.01)	1.62 ± 0.31 (1.20–2.10)	1.72 ± 0.31 (1.40–2.20)	1.70 ± 0.20 (1.40–1.90)	1.62 ± 0.41 (1.20–2.10)	1.45 ± 0.22 (1.20–1.70)	*p* > 0.05	N/A
Chloride	mg/l	5.87 ± 2.66 (2.79–9.31)	5.24 ± 0.87 (4.01–6.64)	4.23 ± 2.36 (2.07–8.51)	6.50 ± 2.37 (3.40–9.37)	7.14 ± 3.83 (4.17–14.60)	9.32 ± 3.53 (2.92–12.50)	*p* > 0.05	200.00
Hardness	mg/l	8.07 ± 8.43 (1.20–21.90)	5.09 ± 2.66 (1.84–7.94)	3.84 ± 2.99 (0.60–8.39)	6.54 ± 4.56 (1.09–10.65)	5.57 ± 2.49 (1.21–7.75)	7.09 ± 3.58 (1.07–10.65)	*p* > 0.05	N/A
Nitrate	mg/l	0.01 ± 0.01 (0.00–0.03)	0.03 ± 0.04 (0.01–0.09)	0.03 ± 0.06 (0.00–0.16)	0.03 ± 0.02 (0.00–0.05)	0.02 ± 0.03 (0.00–0.08)	0.06 ± 0.11 (0.00–0.29)	*p* > 0.05	10.00
Sulphate	mg/l	0.68 ± 0.58 (0.18–1.43)	0.34 ± 0.29 (0.12–0.88)	0.41 ± 0.72 (0.03–1.87)	0.80 ± 0.53 (0.12–1.23)	0.44 ± 0.45 (0.12–1.30)	0.84 ± 0.51 (0.18–1.23)	*p* > 0.05	500.00
Phosphate	mg/l	0.12 ± 0.13 (0.00–0.33)	0.03 ± 0.01 (0.00–0.04)	0.04 ± 0.04 (0.00–0.12)	0.30 ± 0.41 (0.01–0.83)	0.20 ± 0.32 (0.01–0.84)	0.24 ± 0.22 (0.05–0.62)	*p* > 0.05	< 5.00
Calcium	mg/l	1.01 ± 0.76 (0.14–2.13)	0.71 ± 0.49 (0.15–1.29)	0.66 ± 0.50 (0.06–1.15)	0.87 ± 0.83 (0.02–1.87)	0.78 ± 0.39 (0.05–1.04)	0.95 ± 0.65 (0.10–1.75)	*p* > 0.05	N/A
Magnesium	mg/l	0.43 ± 0.44 (0.01–0.96)	0.35 ± 0.34 (0.03–0.82)	0.44 ± 0.34 (0.01–0.74)	0.53 ± 0.47 (0.01–1.06)	0.39 ± 0.27 (0.01–0.72)	0.49 ± 0.38 (0.07–1.04)	*p* > 0.05	N/A
Potassium	mg/l	0.52 ± 0.57 (0.01–1.54)	0.37 ± 0.40 (0.01–1.05)	0.32 ± 0.31 (0.01–0.68)	0.60 ± 0.62 (0.01–1.32)	0.39 ± 0.26 (0.01–0.74)	0.48 ± 0.39 (0.00–1.02)	*p* > 0.05	N/A

Note: *p* < 0.01 – Highly Significant Difference; *p* < 0.05 – Significant Difference; *p* > 0.0 – No Significant Difference

### 2.4 Laboratory procedures: The determinants of turbidity, conductivity, dissolved oxygen

The turbidity values measured in Nephelometric turbidity units (NTU) were determined in the laboratory using Hach’s turbidity meter (Model 2100A). The conductivity given in µs/m was determined at 25°C using Jenway digital TDS meter (Model 4076). The dissolved oxygen was determined using the Winkler’s method APHA. In the laboratory, the precipitate formed by addition of winkler solution A and B was dissolved by adding 2ml of concentrated sulphuric acid in the laboratory. Then 100ml of the sample solution was measured and 2 drops of starch indicator added. The dark blue sample solution was titrated to colourless with 0.0125M Thiosulphate.

### 2.5 Air and water temperatures

The mean Air temperatures ranged between 29.52 at Ubogo - 4 and 33.72 ^°^C at Egini - 2. The highest air temperature of 34.20 °C recorded in the month of March was at Ubogo - 4 and lowest at Egini - 1 and Ubogo - 3 respectively with the values of 29.00 °C (Fig. 2). When subjected to Analysis of Variance (ANOVA), there was a high significant difference (*P* < 0.01) between the study stations.

Water temperature showed a similar trend to that of air temperature. The mean values ranged from 25.43 at Ubogo - 4 to 30.43 °C at Egini - 2. Water temperature was lowest (25.10 °C) at Egini - 2 in the month of July and highest (31.20 °C) at Ubogo - 1 in March (Fig. 4). A high significant difference (*P* < 0.01) was observed between the study stations.

### 2.6 Biochemical oxygen demand (BOD_5_) and water quality index (WQI)

The water quality index (WQI) was calculated using the Weighted Arithmetic Index method. The soft nature of the sampled rivers is expected as the corresponding determinant ions were low in this study. There was no significant difference (*P* > 0.05) in the mean BOD_5_ concentrations among the study stations. The mean BOD_5_ concentrations of the surface waters ranged from 1.45 mg/l at Ubogo - 4 to 1.72 mg/l at the Egini - 1 and Ubogo - 1. The lowest BOD_5_ concentrations (1.20 mg/l) were recorded at Egini - 2 in February, Ubogo - 3 in July and Ubogo - 4 in February and April while the highest (2.2 mg/l) was recorded at Ubogo - 1 in the month of June.

### 2.7 Concluding remarks

It is remarked that water quality is a function of either or both natural influences and human activities and its determination involves the spatio-temporal measurements of physico-chemical and biological parameters. Air and water temperatures, TDS, Conductivity and DO were the only physico-chemical parameters that showed significant difference (*P* < 0.05) between the sampled stations. All the rivers sampled in this study were acidic. This is consistent with Nigerian Rivers. The BOD range of 1.20–2.20 mg/l recorded in the above study corroborates the observation that all the rivers sampled witnessed few anthropogenic activities. All the rivers assessed in this study were classified ‘soft’ because they fell below the 50 mg/l set for soft waters. The soft nature of the sampled rivers is unsurprising as the corresponding determinant ions were low in this study.
